# *dSmad2* differentially regulates dILP2 and dILP5 in insulin producing and circadian pacemaker cells in unmated adult females

**DOI:** 10.1371/journal.pone.0280529

**Published:** 2023-01-23

**Authors:** Samuel L. Goldsmith, Stuart J. Newfeld

**Affiliations:** School of Life Sciences, Arizona State University, Tempe, AZ, United States of America; University of Mississippi, UNITED STATES

## Abstract

Much is known about environmental influences on metabolism and systemic insulin levels. Less is known about how those influences are translated into molecular mechanisms regulating insulin production. To better understand the molecular mechanisms we generated marked cells homozygous for a null mutation in the Drosophila TGF-β signal transducer *dSmad2* in unmated adult females. We then conducted side-by-side single cell comparisons of the pixel intensity of two Drosophila insulin-like peptides (dILP2 and dILP5) in *dSmad2*^*-*^ mutant and wild type insulin producing cells (IPCs). The analysis revealed multiple features of *dSmad2* regulation of dILPs. In addition, we discovered that dILP5 is expressed and regulated by *dSmad2* in circadian pacemaker cells (CPCs). Outcomes of regulation by *dSmad2* differ between dILP2 and dILP5 within IPCs and differ for dILP5 between IPCs and CPCs. Modes of *dSmad2* regulation differ between dILP2 and dILP5. *dSmad2* antagonism of dILP2 in IPCs is robust but *dSmad2* regulation of dILP5 in IPCs and CPCs toggles between antagonism and agonism depending upon *dSmad2* dosage. Companion studies of dILP2 and dILP5 in the IPCs of *dCORL* mutant (*fussel* in Flybase and SKOR in mammals) and *upd2* mutant unmated adult females showed no significant difference from wild type. Taken together, the data suggest that *dSmad2* regulates dILP2 and dILP5 via distinct mechanisms in IPCs (antagonist) and CPCs (agonist) and in unmated adult females that *dSmad2* acts independently of *dCORL* and *upd2*.

## Introduction

Cell to cell communication by Transforming Growth Factor-β (TGF-β) proteins mediates countless developmental and homeostatic processes in bilaterian organisms. Disruptions to TGF-β signaling in humans frequently lead to birth defects and disease. A birth defect example is a missense mutation in a TGF-β receptor that causes fibrodysplasia ossificans progressiva, an inherited syndrome characterized by heterotopic bone formation [1]. A disease example is the regulation of adult blood pressure where the loss of TGF-β signaling causes primary arterial hypertension [2]. To better understand TGF-β signaling, powerful genetic approaches in the model organism *Drosophila melanogaster* have been essential for unraveling mechanisms of pathway regulation and function.

A recent analysis in Drosophila showed that unmated adults of both sexes, homozygous for a small deletion that included *dCORL* were missing a distinct subset of IPCs [3]. Interestingly, this defect was rescued by mating. Based on previous genetic analyses in the larval brain and biochemical assays showing that SKOR1 binds the TGF-β signal transducer Smad3 [4], the *dCORL* mutant IPC phenotype implicated TGF-β signaling via *dSmad2* in the regulation of homeostatic adult dILP production.

The Drosophila genome has eight insulin-like peptides. Three of them (dILP2/3/5) are produced in IPCs. These are neurons that form in the embryo and secrete dILPs throughout the lifecycle [5]. IPC development and developmental regulation of dILP expression have been extensively studied. For example, during development activation of dILP2/3/5 requires the transcription factors *eyeless* and *dachshund* [6]. In another example, larvae mutant for the TGF-β family member *dawdle* show increased dILP2 and dILP5 in IPCs [7].

While much is known about environmental influences on metabolism and global insulin levels in Drosophila, less is known about how those influences are translated mechanistically in adult IPCs into dILP regulation. One set of studies identified a role for the Leptin analog *upd2* in the secretion of dILP2/5 in adult males [8–10]. Whether *upd2* regulation of insulin secretion cooperates with TGF-β signaling is unknown.

To clarify the role of TGF-β signaling in dILP regulation, we analyzed dILP2 and dILP5 in marked *dSmad2* mutant IPC clones in unmated adult females. This approach allowed direct comparison to *dCORL* for testing the hypothesis that *dSmad2* and *dCORL* cooperate in the regulation of dILP2 in IPCs. To test the hypothesis of coordinated TGF-β signaling and *upd2* regulation of dILP2 and dILP5 in adult IPCs, we compared the *dSmad2* mutant results with data from *upd2* mutant unmated adult females.

During these studies we discovered that dILP5 is also expressed in circadian pacemaker cells (CPC). We included CPCs in the clonal analysis and found that the outcome of regulation by *dSmad2* differs between dILP2 and dILP5 in IPCs as well as for dILP5 between IPCs and CPCs. The data also showed that modes of regulation by *dSmad2* differ between dILP2 and dILP5 with antagonism of dILP2 in IPCs being robust but regulation of dILP5 in IPCs and CPCs toggling between antagonism and agonism depending upon *dSmad2* dosage. Companion studies of dILP2 and dILP5 in genomic *dCORL* and *upd2* mutant unmated adult female IPCs revealed no changes in dILP2 or dILP5. Taken together, the data suggest that *dSmad2* regulates dILP2 and dILP5 via distinct mechanisms in IPCs (antagonist) and CPCs (agonist) and in adult females that *dSmad2* acts independently of *dCORL* and *upd2*.

## Materials and methods

### Drosophila stocks

CPC marker: *w; P{y[+t7*.*7] w[+mC] = GMR61G12*.*GAL4}attP2* (BDSC #41286). *dSmad2* mutant MARCM clones: *yw P{w[+mC] = UAS*.*mCD8*.*GFP*.*L}Ptp4E*^*LL4*^ *dSmad2*^*MB388*^ *P{ry[+t7*.*2] = neoFRT}19A / FM7C* referred to as *dSmad2* mutant [11] (BDSC #44384), *w P{ry[+t7*.*2] = hs*.*FLP}1 P{w[+mC] = tubulinP*.*GAL80}LL1 P{ry[+t7*.*2] = neoFRT}19A* referred to as Tub.GAL80 [12] (BDSC #5132) and *w; P{w[+mC] = dCORL*.*GAL4(-)*.*PT} P{w[+mC] = UAS*.*GFP*.*S65T} / SM6A* referred to as dCORL.GAL4 [13]. Rescue of *dSmad2* mutant MARCM clones with UAS.*dSmad2*: *dSma*d2 mutant, Tub.GAL80, dCORL.GAL4, and *yw; P{w[+mC] = UAS*.*dSmad2}* referred to as UAS.*dSmad2* [11]. Expression of *dSmad2* RNAi: dCORL.GAL4 and *yw; P{w[+mC] = UAS*.*dSmad2 RNAi} P{w[+mC] = UAS*.*dicer2} / TM6* referred to as UAS.*dSmad2* RNAi [14]. Rescue of *dSmad2* RNAi with UAS.*dSmad2*: dCORL.GAL4, UAS.*dSmad2* RNAi and UAS.*dSmad2*.

*dCORL* RNAi, *upd2Δ* and *Df(4)dCORL*: Parental stocks for genotypes containing various combinations of OK107.GAL4 (BDSC #854), UAS.*dCORL* RNAi, UAS.lacZ and dCORL.AH.lacZ are described in [3]. The deletions *w upd2Δ* (BDSC #55727) and *yw; Df(4)dCORL* were originally reported in [4, 15], respectively.

Stock backgrounds contained either a *white* mutation or *yellow* and *white* mutations as noted above. Stocks were maintained in four narrow glass vials with roughly 5ml of dextrose media each, in an incubator maintaining 25°C with 68% humidity. Media was prepared in 40 bottle batches as follows: agar 20g, yeast 69g, cornmeal 130g, anhydrous dextrose 275g in 2125ml water plus 29ml Tegosept (100 g/l in 95% ethanol). In preparation for experiments, stocks were grown up in six ounce polyethylene bottles for virgin female collection. Collected females were maintained at 18°C with 68% humidity until needed for crosses. Males were taken from stock bottles maintained at 25°C for crosses.

### Drosophila genetics

*dSmad2* mutant MARCM clones and clone rescue with UAS.*dSmad2*: The experimental genotype for *dSmad2* mutant MARCM clones was *dSmad2*^*MB388*^ */* Tub.GAL80; dCORL.GAL4/*+*. The complete genotype was *yw P{w[+mC] = UAS*.*mCD8*.*GFP*.*L}Ptp4E*^*LL4*^ *dSmad2*^*MB388*^ *P{ry[+t7*.*2] = neoFRT}19A / w P{ry[+t7*.*2] = hs*.*FLP}1 P{w[+mC] = tubulinP*.*GAL80}LL1 P{ry[+t7*.*2] = neoFRT}19A; P{w[+mC] = dCORL*.*GAL4(-)*.*PT} P{w[+mC] = UAS*.*GFP*.*S65T}/+*. The experimental genotype for rescued *dSmad2* mutant MARCM clones was *dSmad2*^*MB388*^ */* Tub.GAL80; dCORL.GAL4 */* UAS.*dSmad2*. The complete genotype was *yw P{w[+mC] = UAS*.*mCD8*.*GFP*.*L}Ptp4E*^*LL4*^ *dSmad2*^*MB388*^ *P{ry[+t7*.*2] = neoFRT}19A / w P{ry[+t7*.*2] = hs*.*FLP}1 P{w[+mC] = tubulinP*.*GAL80}LL1 P{ry[+t7*.*2] = neoFRT}19A; P{w[+mC] = dCORL*.*GAL4(-)*.*PT} P{w[+mC] = UAS*.*GFP*.*S65T} / P{w[+mC] = UAS*.*dSmad2}*. The control genotype in the same brain did not express UAS.*dSmad2*. Crosses were maintained at 25°C. Embryos from these crosses aged 4 to 14 hours were heat-shocked for 1 hour at 37°C (to express FLP recombinase initiating mitotic recombination), allowed to recover at 18°C for 1 hour, then returned to 25°C to eclosion.

*dSmad2* RNAi and RNAi rescue with UAS.*dSmad2*: The experimental genotype for *dSmad2* RNAi in IPCs was dCORL.GAL4/+; UAS.*dSmad2* RNAi*/+*. The complete genotype was *yw*/*w*; *P{w[+mC] = dCORL*.*GAL4(-)*.*PT} P{w[+mC] = UAS*.*GFP*.*S65T}/+; P{w[+mC] = UAS*.*dSmad2 RNAi} P{w[+mC] = UAS*.*dicer2}/+*. The control genotype in the same brain did not express UAS.*dSmad2* RNAi. The experimental genotype for rescued *dSmad2* RNAi was dCORL.GAL4 / UAS.*dSmad2;* UAS.*dSmad2* RNAi*/+*. The complete genotype was *yw*/*w*; *P{w[+mC] = dCORL*.*GAL4(-)*.*PT} P{w[+mC] = UAS*.*GFP*.*S65T} / P{w[+mC] = UAS*.*dSmad2}; P{w[+mC] = UAS*.*dSmad2 RNAi} P{w[+mC] = UAS*.*dicer2}/+*. The control genotype in the same brain did not express UAS.*dSmad2*.

*dSmad2* overexpression: The experimental genotype for UAS.*dSmad2* overexpression was UAS.*dSmad2 /* dCORL.GAL4. The complete genotype was *yw*/*w*; *P{w[+mC] = dCORL*.*GAL4(-)*.*PT} P{w[+mC] = UAS*.*GFP*.*S65T} / P{w[+mC] = UAS*.*dSmad2}*. The control genotype in the same brain did not express UAS.*dSmad2*.

To maintain a common culture density across experiments, each cross was conducted with 20 females and 10 males in a glass vial. The vial was passed after four days, then the second vial passed after four days and the adults cleared from the third vial after four days. All vials contained dextrose media in an incubator maintaining 25°C with 68% humidity. Just prior to eclosion of the first flies in each cross, the vials were moved to 18°C with 68% humidity overnight. The next morning virgin females were collected from 18°C between 10:00AM and 12:00PM. The cross was placed back at 18°C, and females of the appropriate genotype identified from those collected. These females were placed, as an age matched group, into glass vials in the 25°C incubator. After 24 hours their brains were dissected and brains in good condition were fixed then stored at -20°C in methanol. The process was repeated until roughly 20 fixed brains were obtained. During each experiment the lab environment provided a roughly 9 hour light and 15 hour dark cycle. This cycle was not a formal variable as light was ambient and dark was not total. In every experiment mutant and wild type cells were from the same brain. The compiled pairs of mutant and wild type cells analyzed for each experiment shared genetic background, cell lineage and environment (including light:dark cycle) within the two hour collection window.

*dCORL* RNAi and *Df(4)dCORL*: Original confocal images of 1 day old unmated adult female IPCs previously assayed by counting dILP2 expressing cells [3] were reanalyzed for single cell dILP2 pixel intensity employing the parameters for *dSmad2* mutant clones. Single cells viewed in a single slice from two pairs of genotypes were compared in FIJI: *Df(4)dCORL* versus dCORL.AH.lacZ and OK107.GAL4 driven UAS.*dCORL* RNAi versus OK107.GAL4 driven UAS.lacZ, with the lacZ genotype in both pairs serving as wild type.

*upd2*Δ: 1 day old unmated adult females and males were analyzed side by side with *w*^*1118*^ serving as wild type. Flies were dissected, fixed, stained with dILP2 and dILP5, then imaged as described [10]. Single cell pixel intensity comparisons in FIJI [16] followed the parameters employed in the analysis of *dSmad2* mutant clones.

### Immunofluorescence

One day old unmated female adult brains were dissected, fixed, stained for GFP, FasII, and either dILP2 or dILP5 then mounted and imaged as described [13]. Primary antibodies: DSHB mouse α-FasII 1D4 (RRID: AB_528235), Abcam rabbit α-GFP ab6556 (RRID: AB_305564), Abcam chicken α-GFP ab13970 (RRID: AB_300798), mouse α-dILP2 and rabbit α-dILP5 (Pierre Leopold, Institute Curie, Paris). Secondary antibodies: α-GFP was detected with AlexaFluor488 (green; excitation 495nm, emission 519nm), α-FasII shown as blue was detected with AlexaFluor546 (yellow; excitation 556nm, emission 573nm), α-dILP2 and α-dILP5 were detected with AlexaFluor633 (far-red; excitation 632nm, emission 647nm). Common settings were utilized for all *dSmad2* experiments and each channel was collected sequentially to minimize bleed-through.

All confocal images from the same *dSmad2* experiment were analyzed in FIJI with the same parameters. Mutant and wild type cells were imaged in the same brain then identified as wild type or mutant by the presence or absence of GFP. For analysis we prioritized mutant and wild type pairs where both cells were in a single slice. A single slice was preferred because a 2μm slice is thinner than the diameter of the cell body of a fly brain neuron. When imaging a single slice we were confident of measuring the pixel intensity from a single cell. Single slices chosen for analysis were those that displayed the nucleus of the mutant and wild type cells at their widest point. This guideline ensured that the chosen slice captured the largest area of fluorescence in each cell and that both cells were in the same plane in the same orientation.

For *upd2Δ* and *dCORL* studies of unmated adult females, a single mutant cell from a single slice (following the parameters for *dSmad2* mutant clones) was compared to a single wild type cell in a different brain. Here it was not possible to compare cells in the same brain as the mutations were genomic and not clonal. In both studies mutants and wild type brains were collected, fixed, stained, imaged, and analyzed side by side to maximize confidence in the comparisons. *upd2Δ* unmated males were included since their dILP2 phenotype in IPCs was known [8].

### Statistics

Within an appropriate image (single slice or small stack) each cell to be analyzed was traced at the plasma membrane and their dILP2 or dILP5 pixel intensities (red channel) measured in FIJI. The Mean and Standard Error of the Mean (Mean ± SEM) for the pixel intensities of a set of cells were calculated in Excel (minimum set was six cells). Statistical analyses of pixel intensities from pairs of mutant and wild type cells in each experiment were conducted in Excel.

Two-tailed paired Student’s t-test (also called a type 1 t-test) evaluates whether the mean of the differences for each pair in two groups significantly deviates from zero, the mean of the null hypothesis. The standard probability for significance was applied (p ≥ 0.05). The rationale for employing a type 1 test is that the cells being compared are in the same brain with just a single recombination event separating them genotypically. Their developmental lineage, their cellular environment and their organismal life history are the same. The alternative type 3 test evaluates whether the difference of the means of two groups significantly deviates from zero, the mean of the null hypothesis. To increase confidence, all statistical analyses reported with a type 1 test were repeated with a type 3 test. While the type 3 p-values were not identical, outcomes were unchanged (i.e., no significant differences became not significant and vice versa).

The formula for the illustrative statistic Δ% is clone pixel intensity minus wild type pixel intensity divided by wild type pixel intensity. The sign of the Δ% was positive if clone pixel intensity was more than wild type and negative if the clone was less. Note that Δ% shown in figures provides a measure of the size and sign of the difference in pixel intensity between the individual mutant and wild type cells shown.

## Results

### dILP5 is coexpressed with Pigment dispersing factor (Pdf) in circadian pacemaker cells

**Fig 1:**
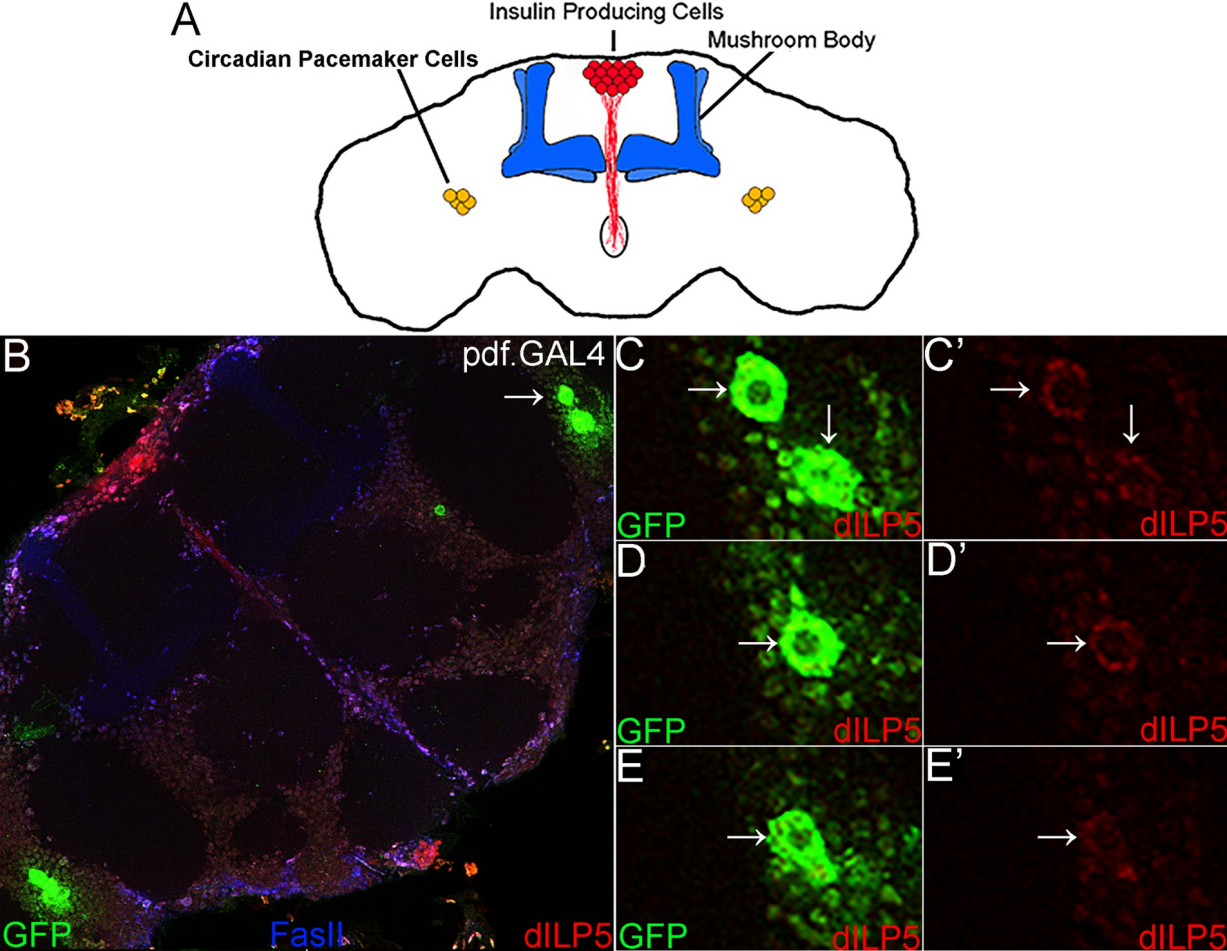
dILP5 is coexpressed with Pdf.*GAL4* in circadian pacemaker cells. A) Illustration of a Drosophila adult brain in anterior view indicating clusters of insulin producing cells (IPCs; red express dILP2 and dILP5), circadian pacemaker cells (CPCs; yellow express dILP5), and the mushroom body (blue; for orientation). B-E) Brain in anterior view from a one day old unmated adult female with Pdf.GAL4 driven UAS.GFP. The secreted neuropeptide Pdf is a marker for small ventral Lateral Neurons sLNv also known as circadian pacemaker cells (CPCs; [18]). Pdf.GAL4 neurons express GFP (green) and dILP5 (red). B) Stack of CPCs (8 slices). Arrow indicates one of the two symmetrical clusters of CPCs that coexpress Pdf.GAL4 and dILP5. C-E) Single confocal slices of the indicated cluster. Two color images on the left and the red channel (dILP5) on the right. Slices together show that all four CPCs in the cluster coexpress Pdf.GAL4 and dILP5.

dCORL.GAL4 was shown to be expressed in a subset of IPCs, the mushroom body and four pairs of neurons in a ventral lateral region of the central brain that also express dILP5 (Fig 1A; [13]). We previously thought the lateral cells were located in the lobula plate and thus formed part of the visual system. To confirm the identity of the lateral cells, we performed co-expression experiments with dILP5 and GFP from two well-characterized GAL4 drivers. We expected to see co-expression of dILP5 with the visual system line GawB-3A [17]. We chose Pdf.GAL4 expressed in circadian pacemaker neurons (CPCs; also known by their anatomical location—small ventral Lateral Neurons sLNv; [18]) as a negative control. CPCs were considered a negative control because no connection between dILP5 and CPCs was known. Instead IPCs were shown to be electrically connected to a distinct set of circadian cells in a dorsal lateral region [19].

We observed that dILP5 (Fig 1B) was co-expressed with Pdf.GAL4 in CPCs. All four cells of the CPC cluster co-express Pdf.GAL4 and dILP5 (Fig 1C–1E). In checking the literature again, we noted studies showing that mammals display a circadian rhythm for glucose metabolism (e.g., [20]), but we could not find any reports of an intracellular connection between the clock and insulin production. The presence of dILP5 in CPCs in unmated adult female brains provides a new avenue for investigation of this connection.

### *dSmad2* mutant clones display increased dILP2 in IPCs and decreased dILP5 in CPCs

To examine the role of *dSmad2* in adult IPCs we analyzed dILP2 expression in *dSmad2* mutant MARCM clones. We employed dCORL.GAL4 driven UAS.GFP (together abbreviated as dCORL.GAL4) to identify mutant clones in otherwise phenotypically wild type (genotypically heterozygous) unmated adult females. The absence of haploinsufficiency for *dSmad2* is attested to by its location on the X chromosome where it is hemizygous in all males. The fact that dCORL.GAL4 is expressed in a subset of IPCs [13] and dILP2 is expressed in all IPCs [5] ensures that mutant and wild type cells are present in the same brain, providing a highly rigorous experiment.

We expanded the analysis to include dILP5 in IPCs and CPCs as dCORL.GAL4 is able to identify *dSmad2* mutant MARCM clones in CPCs. We added *dSmad2* RNAi to the study as a second loss of function approach. To confirm the results of the MARCM and RNAi experiments we conducted UAS.*dSmad2* rescue of both phenotypes. This set of four loss of function studies was complemented by a gain of function (overexpression) experiment. Together the five experiments revealed distinct outcomes and modes of *dSmad2* regulation of dILP2 and dILP5.

**Fig 2:**
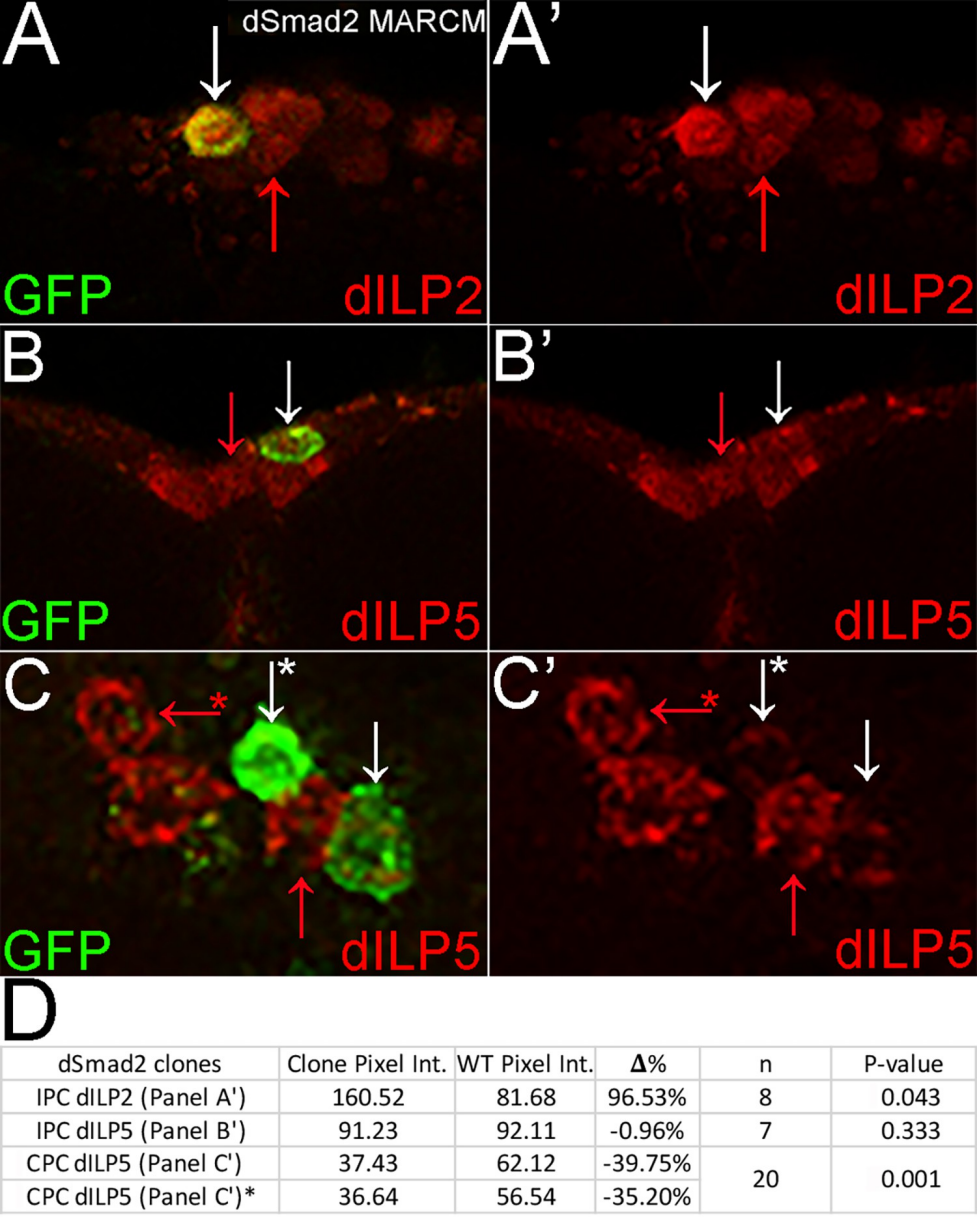
*dSmad2* mutant clones display increased dILP2 in IPCs and decreased dILP5 in CPCs. A-C) Brains in anterior view from one day old unmated adult females with the *dSmad2* mutant MARCM clone genotype. Homozygous *dSmad2* clones express GFP (green) in IPCs and CPCs. IPCs are visualized with dILP2 or dILP5 (red as indicated) and CPCs with dILP5 (red). Left side two color and right side the red channel (dILPs). Paired cells for pixel intensity comparisons are indicated by arrows with white indicating a mutant and red a wild type IPC or CPC. A,A’) Single slice of IPCs. *dSmad2* mutant IPC clone displays significantly increased dILP2 compared to wild type. B,B’) Single slice of IPCs. *dSmad2* mutant IPC clone displays wild type dILP5 expression. C,C’) Stack of CPCs (4 slices). *dSmad2* mutant CPC clones display significantly reduced dILP5 expression compared to wild type. Two comparisons are illustrated with an asterisk marking one pair. D) Table summarizing *dSmad2* mutant clone pixel intensity comparisons in IPCs and CPCs. Note p-values do not refer to Δ% (an illustrative value for the cells compared in each panel), but are derived from a type 1 t-test of the mean of the differences between pairs of wild type and mutant cells shown in Table 1.

Homozygous mutant MARCM clones were generated in the IPCs of unmated adult females heterozygous for *dSmad2*^*MB388*^ (a null allele in the protein-protein interaction domain due to a charge altering mutation in a universally conserved residue E300K; [11]). *dSmad2* mutant IPC clones displayed a significant increase in dILP2 expression compared to adjacent wild type cells (paired t-test of wild type and mutant cells in the same brain and as often as possible the same confocal slice; p = 0.043 see Table 1 for mean ± SEM; Fig 2A and 2D). In contrast, *dSmad2* mutant IPC clones displayed no significant difference in dILP5 expression (p = 0.333; Table 1, Fig 2B and 2D). In contrast to dILP5 IPC clones, *dSmad2* mutant CPC clones displayed a significant decrease in dILP5 expression (p = 0.001; Table 1, Fig 2C and 2D). Note p-values in figure tables do not refer to Δ% (an illustrative value for each panel reflecting the comparison of the two cells shown). Instead p-values derive from a type 1 Student’s t-test of significance for the Mean of the differences between pairs of wild type and mutant cells (Mean ± SEM shown in Table 1).

**Table 1:** Summary of mutant phenotypes for dILP2 & dILP5 pixel intensity in unmated adult female IPCs and CPCs.

Experiment	# brains	# pairs	Experimental Mean ± SEM	WT	Experimental Phenotype	p =
				Mean ± SEM		
*dSmad2* MARCM						Fig 2
IPC dILP2	7	8	97.48 ± 16.10	73.88 ± 9.46	increase	0.043
IPC dILP5	5	7	81.17 ± 12.48	100.04±14.25	wild type	0.333
CPC dILP5	17	20	66.71 ± 8.64	81.55 ± 8.00	decrease	0.001
*dSmad2* MARCM Rescue						S1 Fig in S1 File
IPC dILP2	6	7	95.05 ± 15.07	99.34 ± 9.08	wild type	0.671
IPC dILP5	5	6	63.42 ± 24.44	84.05 ± 31.86	wild type	0.375
CPC dILP5	8	19	70.61 ± 12.00	69.38 ± 8.22	wild type	0.106
*dSmad2* RNAi						Fig 3
IPC dILP2	7	14	54.68 ± 6.06	40.32 ± 4.68	increase	0.013
IPC dILP5	6	16	61.65 ± 5.36	69.79 ± 6.15	wild type	0.116
CPC dILP5	6	7	60.72 ± 4.10	39.24±2.41	increase	0.004
*dSmad2* RNAi Rescue						S2 Fig in S1 File
IPC dILP2	6	17	50.39 ± 4.79	51.41 ± 4.47	wild type	0.661
IPC dILP5	7	21	71.9 ± 6.85	62.23 ± 5.82	increase	0.005
CPC dILP5	6	6	74.85 ± 9.80	56.61 ± 6.34	wild type	0.154
*dSmad2* Overexpression						Fig 4
IPC dILP2	6	16	35.63 ± 2.76	49.16 ± 4.52	decrease	0.003
IPC dILP5	11	31	75.78 ± 7.37	77.85 ± 6.32	wild type	0.607
CPC dILP5	8	12	18.43 ± 5.32	15.70 ± 4.53	increase	0.008
*Df(4)dCORL*						S3 Fig in S1 File
IPC dILP2	4	12	144.75 ± 14.59	118.81±22.41	wild type	0.344
*dCORL* RNAi						S4 Fig in S1 File
IPC dILP2	5	18	125.06 ± 12.87	120.29±11.64	wild type	0.785
*upd2Δ* males						Fig 5
IPC dILP2	10	20	90.73 ± 10.34	49.56 ± 6.60	increase	0.001
IPC dILP5	10	20	64.93 ± 7.99	38.44 ± 3.98	increase	0.002
*upd2Δ* females						
IPC dILP2	12	20	100.29 ± 13.06	74.43 ± 12.97	wild type	0.179
IPC dILP5	12	20	79.6 ± 10.32	59.56 ± 8.73	wild type	0.129

The results from rescue experiments were consistent with the MARCM data providing additional confidence. UAS.*dSmad2* rescued both mutant phenotypes. For dILP2 in IPC clones, UAS.*dSmad2* rescued overexpression to wild type (p = 0.671; Table 1, S1A, S1D Fig in S1 File). For dILP5 in IPC clones, UAS.*dSmad2* had no effect with all cells remaining wild type (p = 0.375; Table 1, S1B, S1D Fig in S1 File). For dILP5 in CPC clones, UAS.*dSmad2* rescued reduced expression to wild type (p = 0.106; Table 1, S1C, S1D Fig in S1 File).

In summary, *dSmad2* mutant MARCM clones reveal two types of *dSmad2* differential regulation of dILP2 and dILP5. The first difference is in IPCs between dILP2 (increased expression revealing a loss of negative regulation) and dILP5 (no difference). The second distinction is between dILP5 in IPCs (no difference) and in CPCs (decreased expression revealing a loss of positive regulation). The rescue of both mutant phenotypes with UAS.*dSmad2* reinforces their connection to *dSmad2* rather than a second mutation distal to the FRT.

### *dSmad2* RNAi matches mutant clones for dILP2 but suggests dosage effects for dILP5

**Fig 3:**
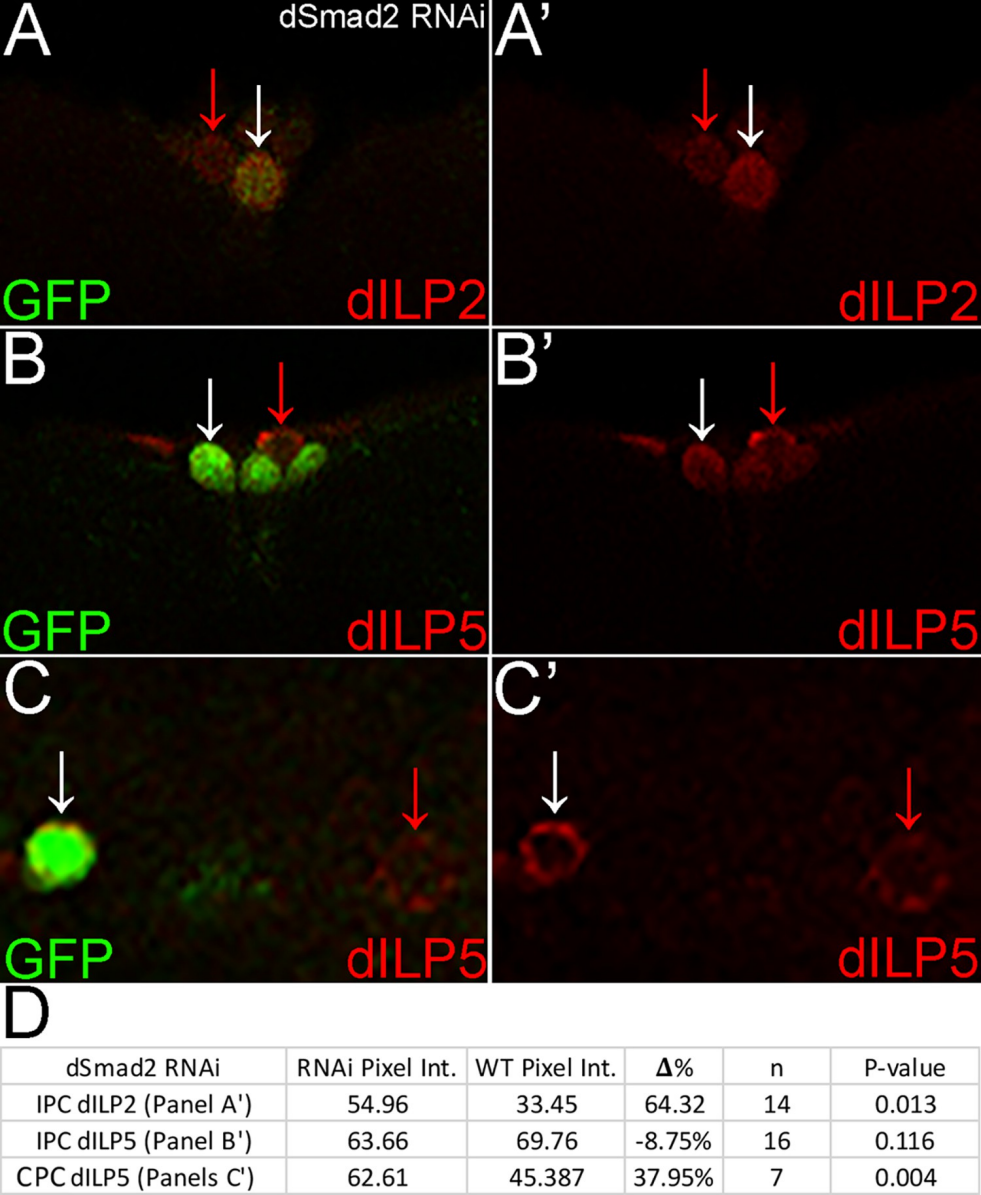
*dSmad2* RNAi matches mutant clones for dILP2 but suggests a dosage effect for dILP5 in CPCs. A-C) Brains as shown in Fig 2 with dCORL.GAL4 driving UAS.*dSmad2* RNAi and UAS.GFP. A,A’) Single slice of IPCs. *dSmad2* RNAi IPC displays significantly increased dILP2 expression compared to wild type. B,B’) Single slice of IPCs. *dSmad2* RNAi IPC displays wild type dILP5 expression C,C’) Single slice of CPCs. *dSmad2* RNAi CPC displays significantly increased dILP5 expression compared to wild type. D) Table as described in Fig 2 summarizing *dSmad2* RNAi pixel intensity comparisons in IPCs and CPCs.

To further support our findings we expressed UAS.*dSmad2* RNAi via dCORL.GAL4. In IPCs, *dSmad2* RNAi phenocopied *dSmad2* mutant clones with overexpression of dILP2 (p = 0.013; Table 1, Fig 3A and 3D). In IPCs there continued to be no change in the expression of dILP5 (p = 0.116; Table 1, Fig 3B and 3D). Interestingly, for dILP5 in CPCs there was an increase in expression (p = 0.004; Table 1, Fig 3C and 3D). Distinct phenotypes for a *dSmad2* mutation in a CPC clone and *dSmad2* RNAi in the same cells could have two causes. The first is the influence of *dSmad2* dosage. Complete loss of function in clones could be interpreted by CPCs distinctly from an RNAi induced partial loss of function. The second is the influence of the circadian pacemaker, a variable we did not formally control in these experiments.

We then added UAS.*dSmad2* to the *dSmad2* RNAi genotype for rescue studies. For dILP2 in IPCs, UAS.*dSmad2* rescued overexpression to wild type (p = 0.661; Table 1, S2A, S2D Fig in S1 File). For dILP5 in IPCs, adding UAS.*dSmad2* to the genotype led to an unexpected increase in expression (p = 0.005; Table 1, S2B, S2D Fig in S1 File). However, the increase is consistent with the influence of dosage. Partial loss due to *dSmad2* RNAi is overcompensated with UAS.*dSmad2*, whereas in *dSmad2* mutant clones complete loss was simply compensated. For dILP5 in CPCs, *UAS*.*dSmad2* rescued overexpression to wild type (p = 0.154; Table 1, S2C, S2D Fig in S1 File).

In summary, the *dSmad2* RNAi phenotype for dILP2 in IPCs was consistent with phenotypes for MARCM clones and both rescue studies. The RNAi phenotype for dILP5 in IPCs was consistent with MARCM clones and the MARCM rescue study. The RNAi rescue study suggested a dosage effect for dILP5 in IPCs with expression increasing from wild type to above wild type. For dILP5 in CPCs, the RNAi suggestion of a dosage effect is strengthened by the presence of increased dILP5 versus a decrease in MARCM clones. Rescue of CPC phenotypes with UAS.*dSmad2* reinforces their connection to *dSmad2* rather than a second mutation.

### *dSmad2* overexpression phenotype complements the *dSmad2* loss phenotype

**Fig 4:**
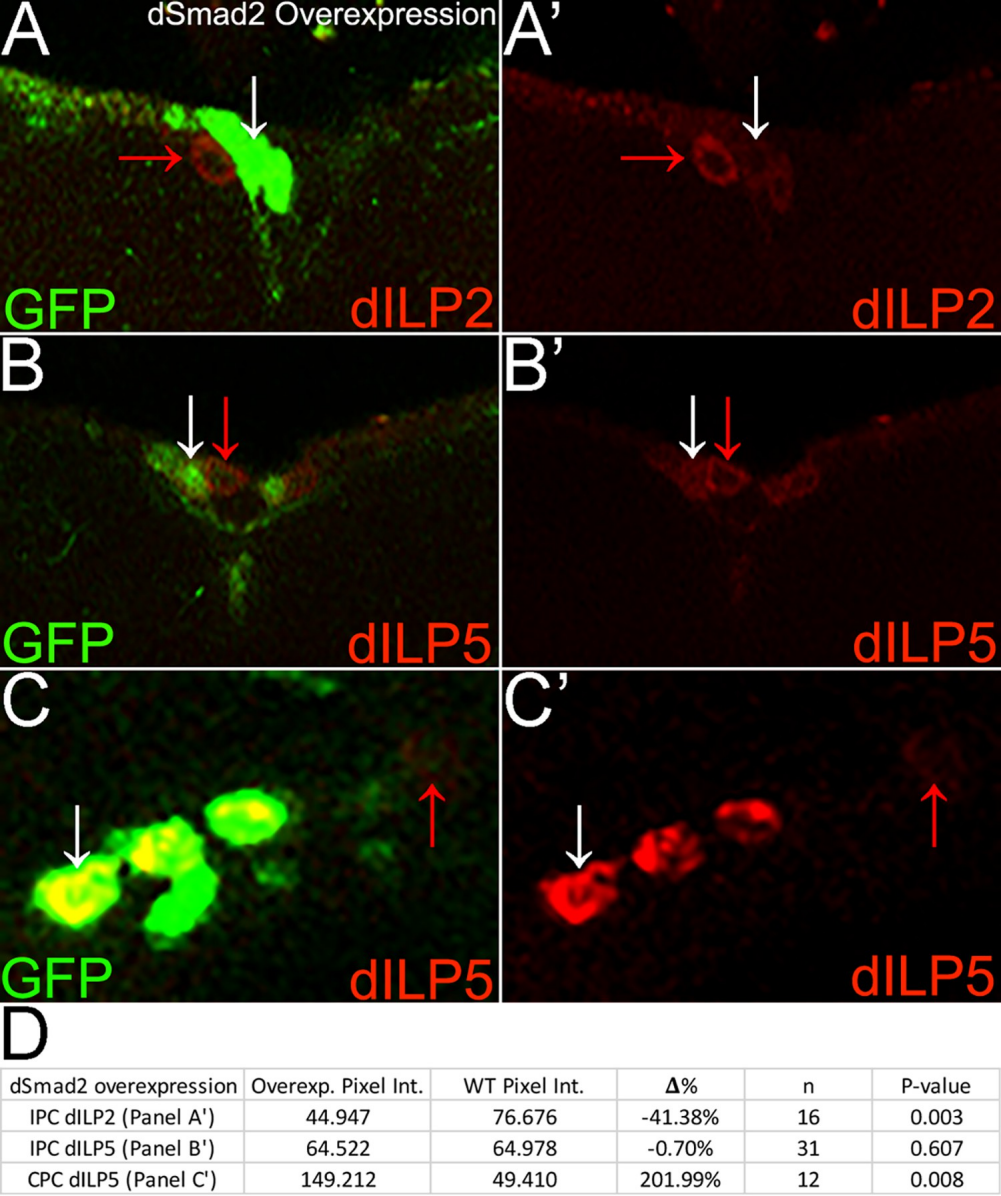
*dSmad2* overexpression phenotype complements the *dSmad2* loss phenotype. A-C) Brains as shown in Fig 2 with dCORL.GAL4 driving UAS.*dSmad2* and UAS.GFP. A,A’) Single slice of IPCs. *dSmad2* overexpressing IPC displays significantly decreased dILP2 expression compared to wild type. B,B’) Single slice of IPCs. *dSmad2* overexpressing IPC displays wild type dILP5 expression. C,C’) Stack of CPCs (2 slices). *dSmad2* overexpressing CPC displays significantly increased dILP5 expression compared to wild type. D) Table as described in Fig 2 summarizing *dSmad2* overexpression pixel intensity comparisons in IPCs and CPCs.

For completeness, we conducted a gain of function study expressing UAS.*dSmad2* in wild type IPCs. This led to dILP2 phenotypes that were consistent with loss of function studies. For dILP2 in IPCs, complementary to the significant increase seen with *dSmad2* loss, expressing UAS.*dSmad2* significantly reduced expression below wild type (p = 0.003; Table 1, Fig 4C and 4D). For dILP5 in IPCs, consistent with *dSmad2* loss, expressing UAS.*dSmad2* had no effect with all cells remaining wild type (p = 0.607; Table 1, S4B, S4D Fig in S1 File). For dILP5 in CPCs, complementary to the significant decrease seen with *dSmad2* loss, expressing UAS.*dSmad2* significantly increased expression above wild type (p = 0.008; Table 1, Fig 4C and 4D).

In summary, all five experiments for dILP2 in IPCs are consistent with *dSmad2* acting as an antagonist (MARCM clones, RNAi, both rescues and overexpression). For dILP5 in IPCs, four of five experiments indicate no role for *dSmad2* in dILP5 IPC regulation. The anomalous result, an increase with *dSmad2* RNAi rescue, we consider evidence of a dosage effect of *dSmad2* on dILP5 IPC expression. For dILP5 in CPCs, four of five experiments indicate *dSmad2* acts as an agonist in dILP5 CPC regulation. The anomalous result, an increase with *dSmad2* RNAi, we consider evidence of a dosage effect of *dSmad2* on dILP5 CPC regulation, though we are unable to rule out an effect of the circadian clock. Regardless of why, the simple regulatory interaction between *dSmad2* and dILP2 stands in contrast to the more complicated interaction between *dSmad2* and dILP5.

### Neither *dCORL* nor *upd2* mutants phenocopy loss of *dSmad2* in adult female IPCs

We then addressed whether *dSmad2* cooperates with *dCORL* and whether *dSmad2* cooperates with *upd2* in IPCs of unmated adult females. To begin we reanalyzed *Df(4)dCORL* and *dCORL* RNAi images from [3]. The logic was that the impact of a *dCORL* mutation on IPC number, as reported there, is likely independent of *dSmad2* but the remaining IPCs could have increased dILP2 expression resulting from the loss of a second role for *dCORL* in cooperation with *dSmad2*. The reanalysis of both genotypes showed there is no difference in dILP2 expression levels in IPCs between *Df(4)dCORL* and wild type (p = 0.344; Table 1, S3 Fig in S1 File) or between *dCORL* RNAi and wild type (p = 0.785; Table 1, S4 Fig in S1 File). We conclude from these studies that *dCORL* and *dSmad2* function independently upstream of dILP2 in IPCs.

The *dCORL* data also counter an alternative explanation for our *dSmad2* data in IPCs. The alternative posits that reliance on dCORL.GAL4 to identify clones led to an artificial result because dCORL.GAL4 expressing IPCs and dCORL.GAL4 non-expressing IPCs are somehow different in a way that impacts dILP2. Data from S3 and S4 Figs in S1 File showing no effect of *dCORL* mutants (either genomic or RNAi) on dILP2 expression in IPCs reveals that dCORL.GAL4 expressing and non-expressing cells are equivalent with regards to dILP2.

**Fig 5:**
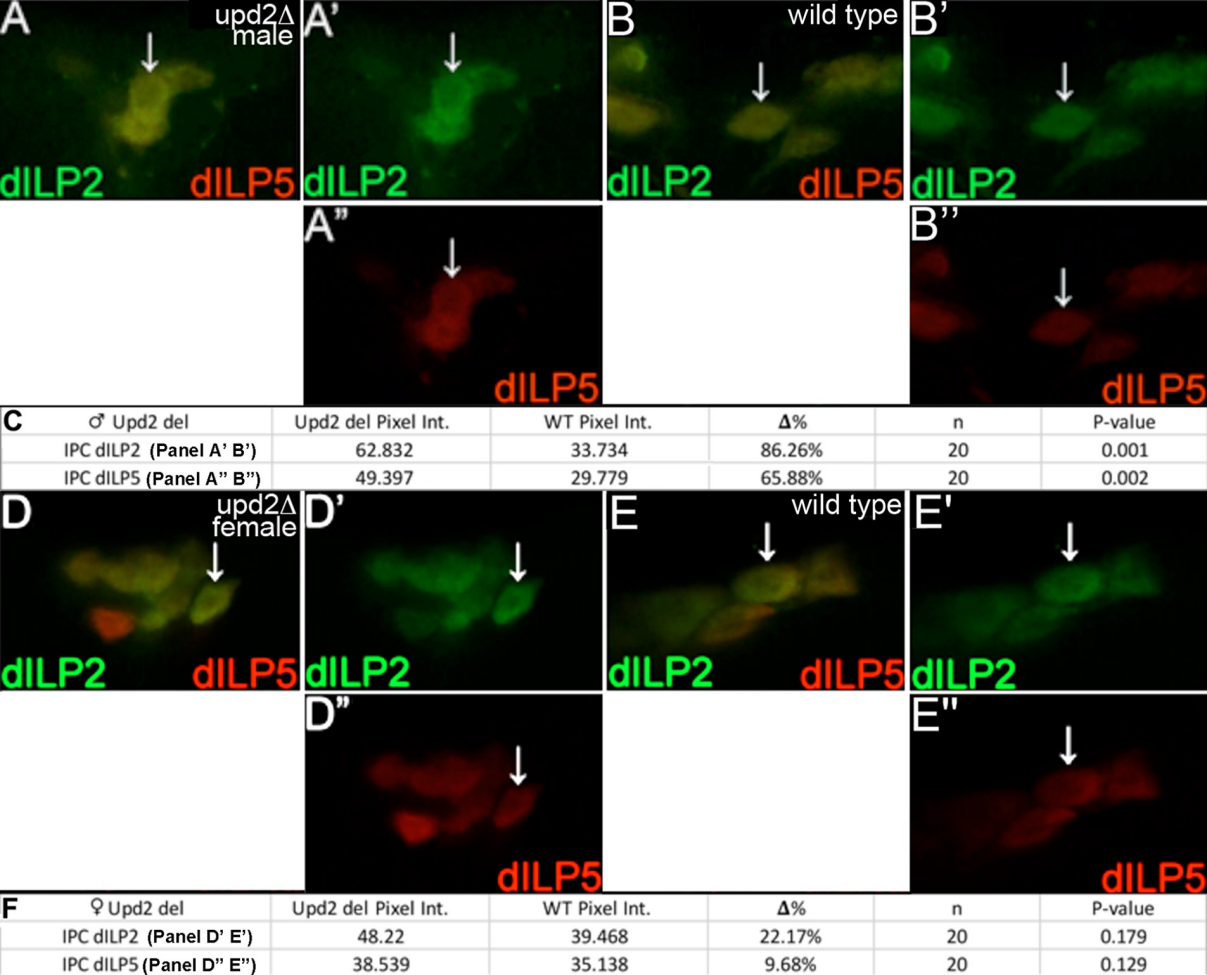
*upd2* mutant unmated adult female IPCs do not phenocopy *dSmad2* mutants. Brains from unmated adult males and females as shown in Fig 2. A-C) One day old unmated adult male brains with IPCs labeled for dILP2 (green) and dILP5 (red). Each panel group shows two colors on the left and each single channel on the right. White arrows indicate IPCs analyzed for pixel intensity. A,A”) *upd2* mutant male IPCs. B,B”) Wild type male IPCs. *upd2* mutant male IPCs display significantly increased dILP2 and dILP5 pixel intensity compared to wild type males. C) Table as described in Fig 2 summarizing *upd2* unmated mutant male pixel intensity comparisons in IPCs. D-F) One day old unmated adult female brains with IPCs labeled as above. D-D”) *upd*2 mutant female IPCs. E-E”) Wild type female IPCs. *upd2* mutant female IPCs display wild type dILP2 and dILP5 expression. F) Table as described in Fig 2 summarizing *upd2* mutant female pixel intensity comparisons in IPCs.

For *upd2*, we examined unmated adult male brains where it has been shown that dILP2 and dILP5 expression is increased in *upd2* mutants [8]. We reproduced that data (dILP2 p = 0.001 and dILP5 p = 0.002; Table 1, Fig 5A, 5B and 5E). We then analyzed unmated adult female brains for dILP2 and dILP5. For both there was a modest increase that was not significant in *upd2* mutants versus wild type (dILP2 p = 0.179 and dILP5 p = 0.129; Table 1, Fig 5C–5E). We conclude that *upd2* does not cooperate with *dSmad2* to regulate dILP2 expression in unmated adult female IPCs. Interestingly, the data suggest that *upd2* regulates dILP2 in IPCs in a gender-specific fashion. For dILP5 in unmated adult female IPCs, loss of neither *dSmad2* nor *upd2* had any significant impact suggesting they are either uninvolved in dILP5 regulation or that additional experiments are needed.

## Discussion

Our analysis of *dSmad2* regulation of dILP2 and dILP5 in unmated adult females led to multiple sets of unexpected observations. The first set of unanticipated findings was the identification of dILP5 expression in CPCs and that dILP5 is positively regulated there by *dSmad2*. The presence of the secreted metabolic hormone dILP5, the secreted pacemaker peptide Pdf and the TGF-β family signal transducer dSmad2 in these cells suggest that CPCs are a hub for responding to environmental influences through altered clock rhythms and systemic insulin levels. For example, environmental signals could be transmitted to CPCs via the starvation sensitive TGF-β family member Dawdle [21]. Once activated by Dawdle signaling, our hypothesis is that dSmad2 directly regulates dILP5 levels and also modulates the clock via interactions with the essential cycling protein Vrille [22]. A potential dSmad2—Vrille interaction in CPCs is consistent with by the discovery of *vrille* in an assay for new TGF-β pathway components [23]. Testing this hypothesis is one avenue for future investigation. Another is testing the hypothesis that the circadian clock may influence dILP5 CPC expression.

A second set of unanticipated findings was two types of differential relationships between *dSmad2*, dILP2 and dILP5. First, *dSmad2* regulatory outcomes (agonist versus antagonist) differed between dILP2 and dILP5 in IPCs and for dILP5 between IPCs and CPCs. For the latter, an alternative explanation is the influence of the circadian pacemaker on dILP5 in CPCs. Second, *dSmad2* regulatory modes (robust versus dosage dependent) differed between dILP2 and dILP5. For dILP5, dosage dependence could be further investigated by examining *dSmad2* transcript levels in CPCs. The data serves as a cautionary tale that emphasizes the importance of cellular context in understanding dILP regulatory interactions.

A third unexpected observation was that *dCORL* and *dSmad2* have independent effects on dILP2 in unmated adult female IPCs. A *dCORL* mutation reduces the number of dILP2 cells but has no effect on dILP2 expression. In contrast, a *dSmad2* mutation increases dILP2 expression. The relationship between *dCORL* and *dSmad2* upstream of dILP2 in unmated adult female IPCs is distinct from their cooperation upstream of EcRB-1 in female 3rd instar larval mushroom bodies [4]. It appears that dCORL is not a "core" signal transducer for TGF-β proteins, but rather a context dependent one similar to dSno (Snoo in Flybase; [24]). Evidence that *dCORL* and *dSmad2* act independently also suggests that dILP2 expression in *dSmad2* mutant clones in mated and unmated adult females will be similar, as the effect of mating on dILP2 was only seen in *dCORL* mutants.

A fourth unexpected result was the absence of any impact on dILP2 or dILP5 in *upd2* mutant unmated adult females. This finding expands the data’s cautionary tale to include organismal context for dILP regulation and reinforces the need for investigation of gender specific developmental and homeostatic mechanisms. With *dSmad2* on the X chromosome and the lethality of *dSmad2* mutants, it is not possible to obtain *dSmad2* mutant males with clones. Thus whether *dSmad2* and *upd2* have complementary gender specificity in the regulation of dILP2 and dILP5 in adult IPCs can only be addressed by the less rigorous RNAi approach, another future direction.

In conclusion, the data suggest that *dSmad2* regulates dILP2 and dILP5 via distinct mechanisms in IPCs (antagonist) and CPCs (agonist) and that *dSmad2* in adult females acts independently of *dCORL* and *upd2*.

## Supporting information

S1 File (PDF)
