# Supplementary material for: dSmad2 differentially regulates dILP2 and dILP5 in insulin producing and circadian pacemaker cells in unmated adult females
Source: PLoS One. 2023 Jan 23;18(1):e0280529. doi: 10.1371/journal.pone.0280529 (PMC9870127; doi:10.1371/journal.pone.0280529)
Supplement: S1 File — (PDF) [file pone.0280529.s001.pdf]

Supplemental Information - Four Figures

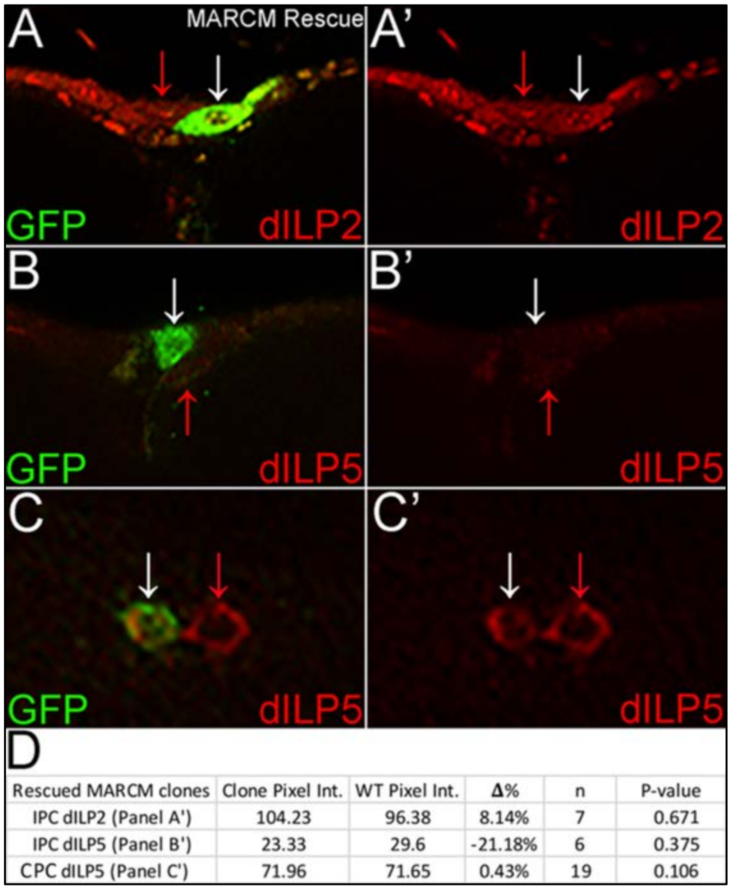

D

Rescued MARCM clones	Clone Pixel Int.	WT Pixel Int.	Δ%	n	P-value
IPC dILP2 (Panel A')	104.23	96.38	8.14%	7	0.671
IPC dILP5 (Panel B')	23.33	29.6	-21.18%	6	0.375
CPC dILP5 (Panel C')	71.96	71.65	0.43%	19	0.106

Fig. S1. Expressing *dSmad2* in *dSmad2* mutant clones rescues mutant phenotypes. A-C) Brains as shown in Fig. 2 with UAS.*dSmad2* added to the MARCM genotype. A,A') Single slice of IPCs. Rescued *dSmad2* mutant IPC clone displays wild type dILP2 expression. B,B') Stack of IPCs (11 slices). Rescued *dSmad2* mutant IPC clone displays wild type dILP5 expression. C,C') Single slice of CPCs. Rescued *dSmad2* mutant CPC clone displays wild type dILP5 expression. D) Table as described in Fig. 2 summarizing rescued *dSmad2* mutant clone pixel intensity comparisons in IPCs and CPCs.

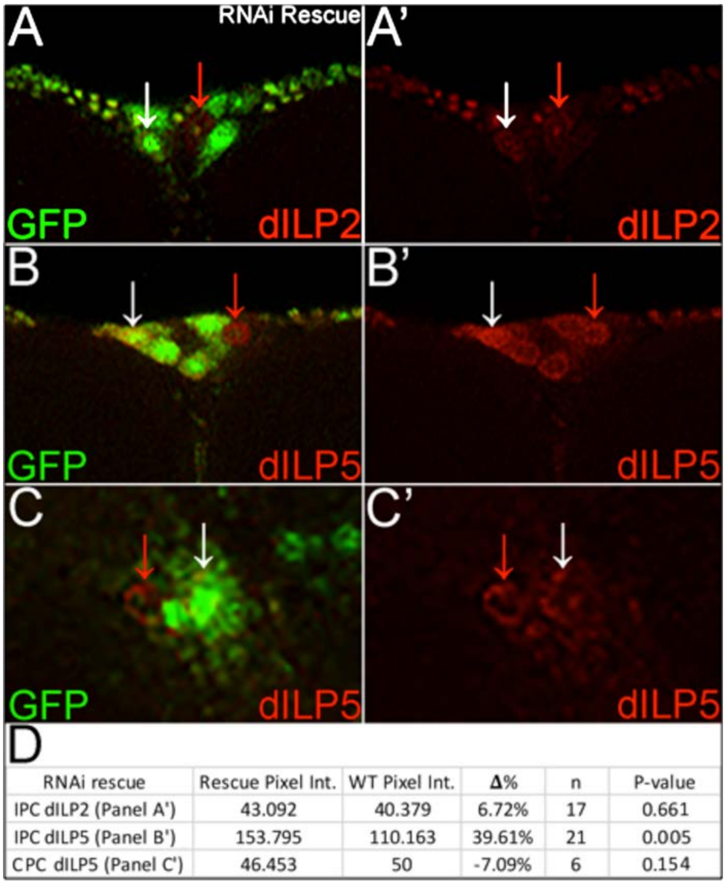

D

RNAi rescue	Rescue Pixel Int.	WT Pixel Int.	Δ%	n	P-value
IPC dILP2 (Panel A')	43.092	40.379	6.72%	17	0.661
IPC dILP5 (Panel B')	153.795	110.163	39.61%	21	0.005
CPC dILP5 (Panel C')	46.453	50	-7.09%	6	0.154

Fig. S2. Coexpression of *dSmad2* and *dSmad2* RNAi rescues mutant phenotypes but suggests a dosage effect for dILP5 in IPCs. A-C) Brains as shown in Fig. 3 with UAS.*dSmad2* added to the UAS.*dSmad2* RNAi genotype. A,A') Single slice of IPCs. Rescued *dSmad2* RNAi IPC displays wild type dILP2 expression. B,B') Single slice of IPCs. Rescued *dSmad2* RNAi IPC displays increased dILP5 expression compared to wild type. C,C') Stack of CPCs (2 slices). Rescued *dSmad2* RNAi CPC displays wild type dILP5 expression. D) Table as described in Fig. 2 summarizing rescued *dSmad2* RNAi pixel intensity comparisons in IPCs and CPCs.

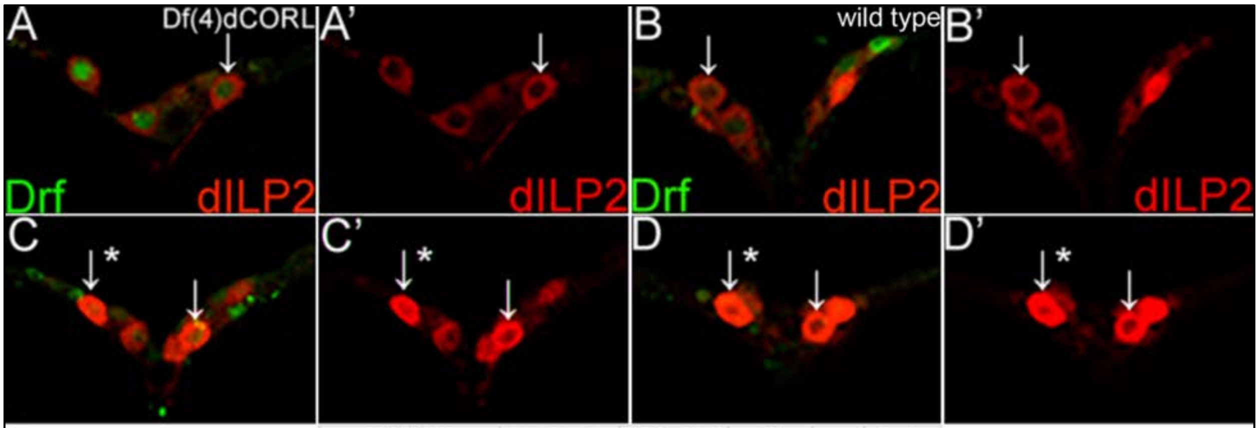

E

Df(4)dCORL	Df(4) Pixel Int.	WT Pixel Int.	Δ%	n	P-value
IPC dILP2 (Panel A'B')	97.133	105.106	-7.59%	12	0.344
IPC dILP2 (PanelC'D')	198.317	216.986	-8.60%		
IPC dILP2 (PanelC'D')*	208.983	243.149	-14.05%		

Fig. S3: *Df(4)dCORL* does not phenocopy loss of *dSmad2* in IPCs. A-D) Reanalyzed brains from Tran et al. (2018) as shown in Fig. 2 in single slices. Females homozygous for the small deletion *Df4(dCORL)* in the left two columns or wild type in the right two columns reflecting the transcription factor Drifter (Drf; green) and dILP2 (red). White arrows in the same row indicate IPCs with their dILP2 pixel intensities (red channel) compared between *Df(4)dCORL* and wild type. Three representative pairs are shown with one in the top row (A' versus B') and two in the second row (asterisk marked cells are one pair and non-asterisk cells the other; C' versus D'). E) Table as described in Fig. 2 summarizing *Df(4)dCORL* pixel intensity comparisons in IPCs.

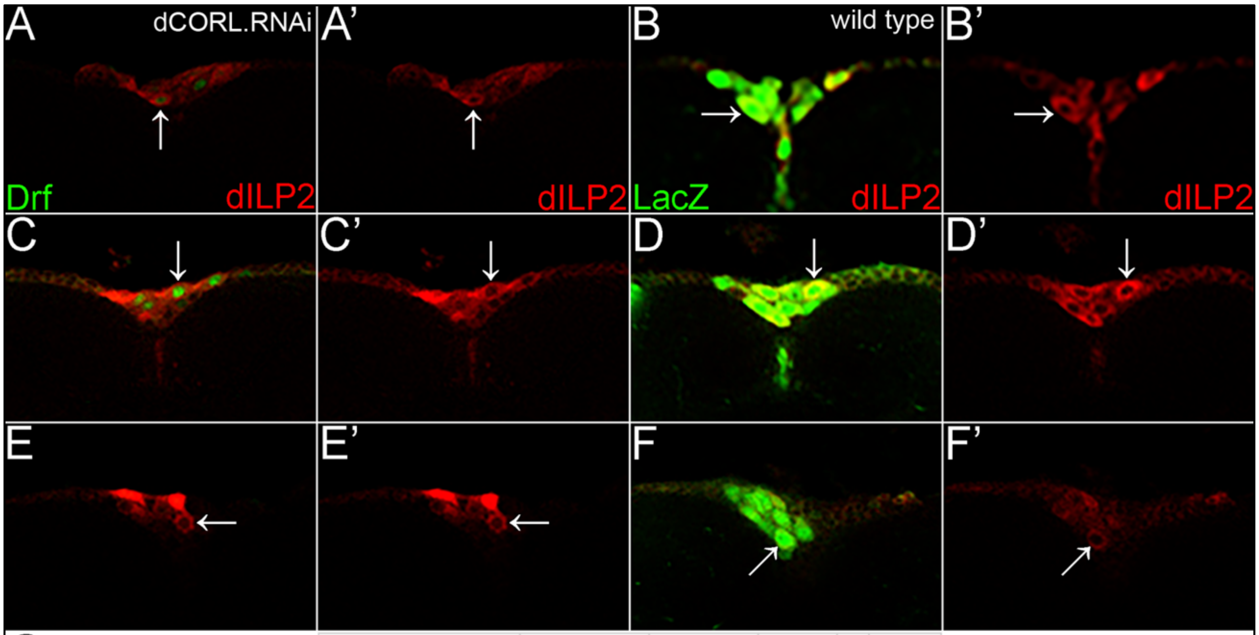

G

dCORL RNAi	RNAi Pixel Int.	WT Pixel Int.	Δ%	n	P-value
IPC dILP2 (Panels A', B')	87.873	94.96	-7.46%		
IPC dILP2 (Panels C', D')	132.62	159.721	-16.97%	18	0.785
IPC dILP2 (Panels E', F')	83.75	50.659	65.32%		

Fig. S4: *dCORL* RNAi does not phenocopy loss of *dSmad2* in IPCs. A-F') Reanalyzed brains from Tran et al. (2018) as shown in Fig. 2 in single slices. Females with OK107.GAL4 driven UAS.*dCORL* RNAi in the left two columns or OK107.GAL4 driven UAS.lacZ in the right two columns serving as wild type. Brains display Drifter (first column) or lacZ (third column) in green with dILP2 in red. White arrows in the same row indicate IPCs with their dILP2 pixel intensities (red channel) compared between UAS.*dCORL* RNAi and wild type dilP2. Three representative pairs are shown with one in each row. G) Table as described in Fig. 2 summarizing *dCORL* RNAi pixel intensity comparisons in IPCs.
